# Supplementary material for: Biochemical Mechanism of Fresh-Cut Lotus (Nelumbo nucifera Gaertn.) Root with Exogenous Melatonin Treatment by Multiomics Analysis
Source: Foods. 2022 Dec 22;12(1):44. doi: 10.3390/foods12010044 (PMC9818798; doi:10.3390/foods12010044)
Supplement: Supplementary file 1 [file foods-12-00044-s001.zip › Supplementary Material.pdf]

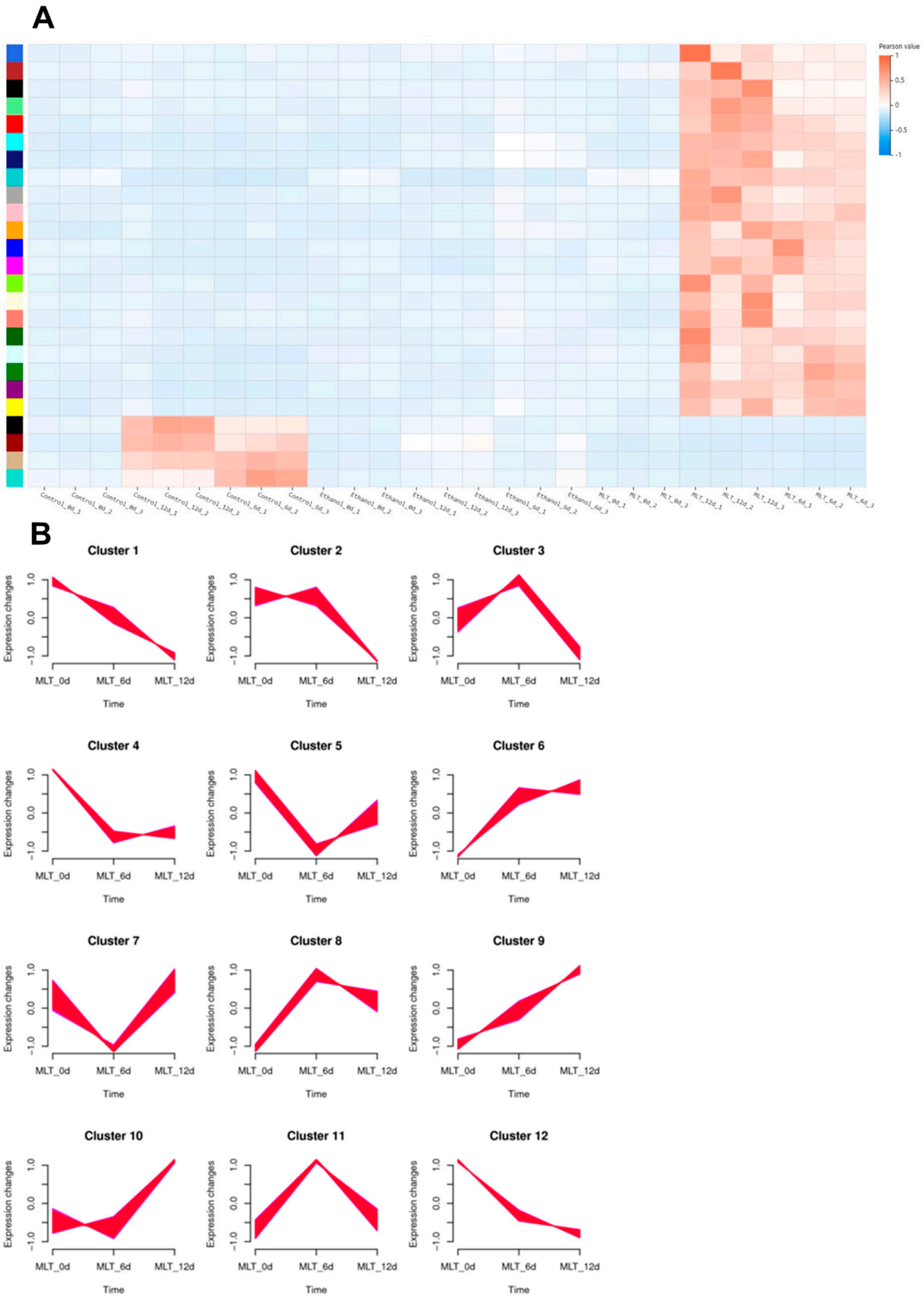
A
Pearson value
1
0.5
0
-0.5
-1
Control_0d_1
Control_0d_2
Control_0d_3
Control_12d_1
Control_12d_2
Control_12d_3
Control_6d_1
Control_6d_2
Control_6d_3
Ethanol_0d_1
Ethanol_0d_2
Ethanol_0d_3
Ethanol_12d_1
Ethanol_12d_2
Ethanol_12d_3
Ethanol_6d_1
Ethanol_6d_2
Ethanol_6d_3
MLT_0d_1
MLT_0d_2
MLT_0d_3
MLT_12d_1
MLT_12d_2
MLT_12d_3
MLT_6d_1
MLT_6d_2
MLT_6d_3
B
Cluster 1
Cluster 2
Cluster 3
Cluster 4
Cluster 5
Cluster 6
Cluster 7
Cluster 8
Cluster 9
Cluster 10
Cluster 11
Cluster 12
Expression changes
1.0
0.0
-1.0
MLT_0d
MLT_6d
MLT_12d
Time

Figure S1. Co-expression network during melatonin treatment. A: WGCNA analysis of RNA-seq data from different stages of melatonin treatment. B: Network hubs regulating genes in differentiation stage. Color codes indicate that the gene displayed with the peak expression in the corresponding stages.

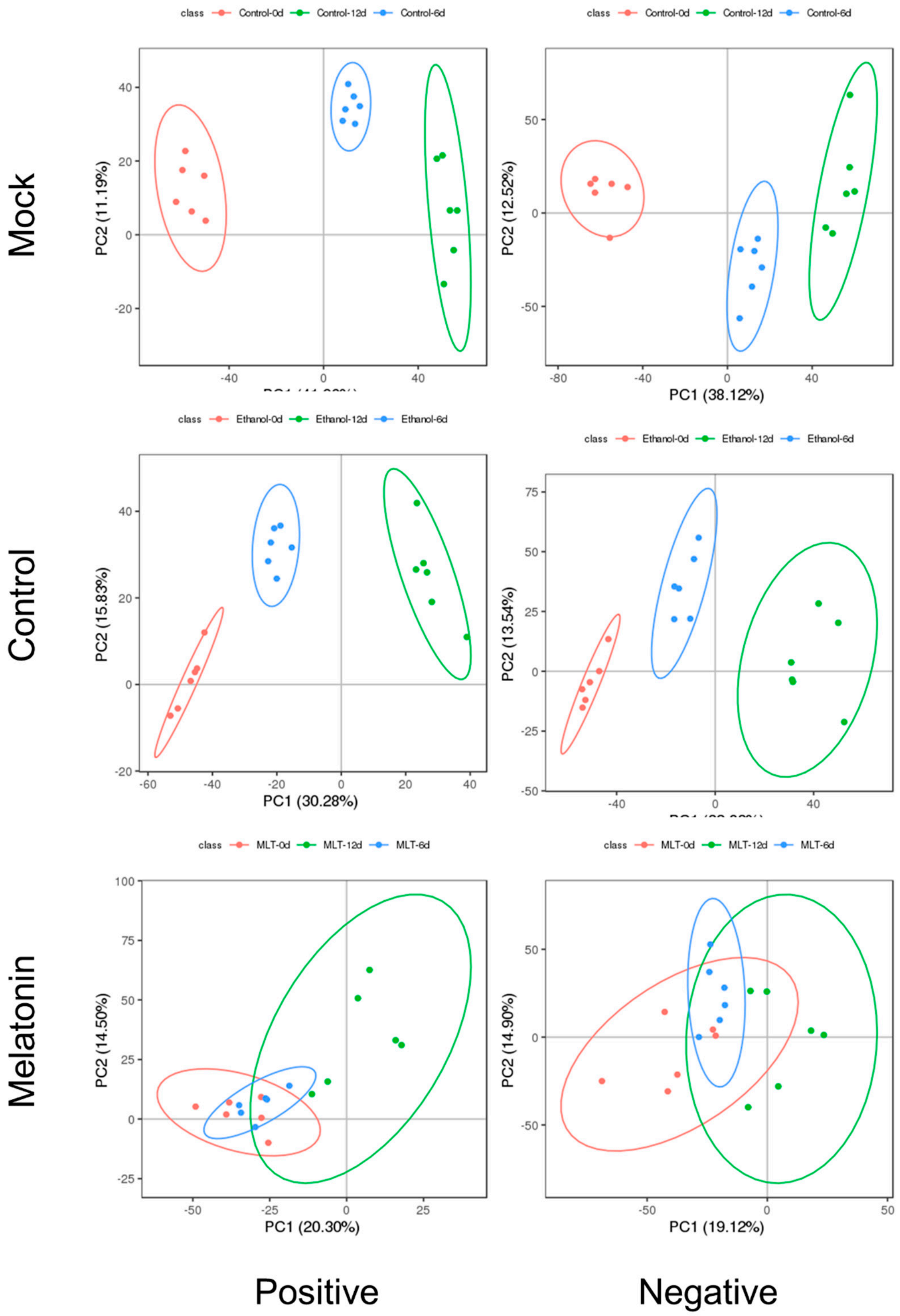

Figure S2. PCA principal component analysis of the expression profiles of fresh cut lotus root were treated with melatonin solution.

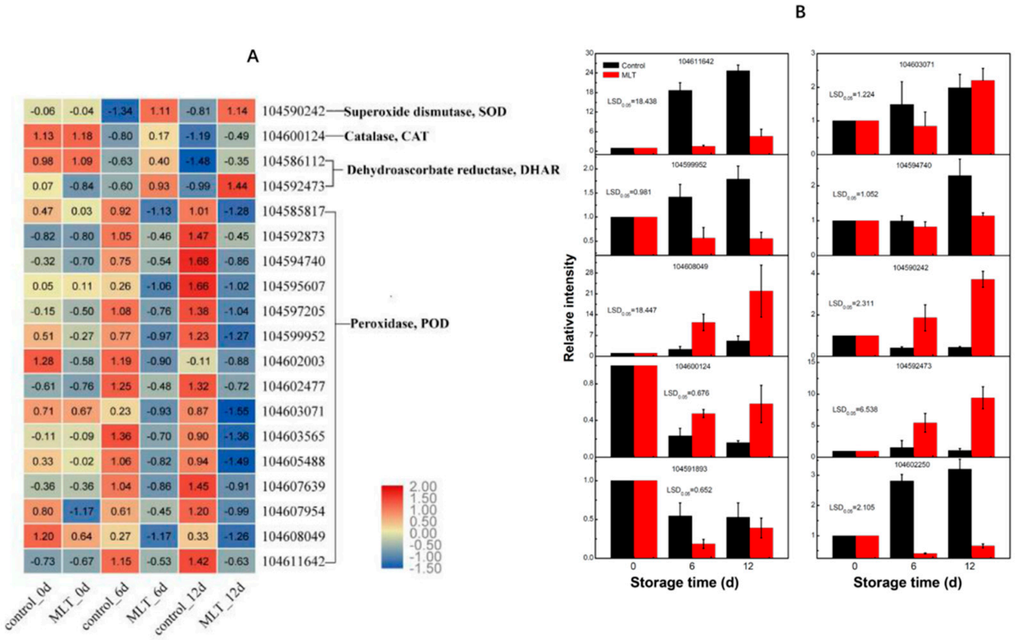

Figure S3. The expression profiles of ROS metabolism-related candidate genes (A) and the qRT-PCR analysis of 10 DEGs (B). Rows and columns in the heatmap indicate candidate genes and samples collected. The color scale at the right indicates the log2 (FPKM) values. CAT, catalase; DHAR, dehydroscorbate reductase; FPKM, expected number of fragments per kilobase of transcript sequence per million base pairs sequenced; POD, peroxidase; SOD, superoxide dismutase. Relative gene expressions were normalized by comparison with the expression of lotus β-actin and analyzed using the $2^{-\Delta\Delta CT}$ Method. The expression values were adjusted by setting the expression of 0d to be 1 for each gene. All qRT-qPCRs for each gene used three biological replicates, with three technical replicates per experiment; the error bars indicate SE.

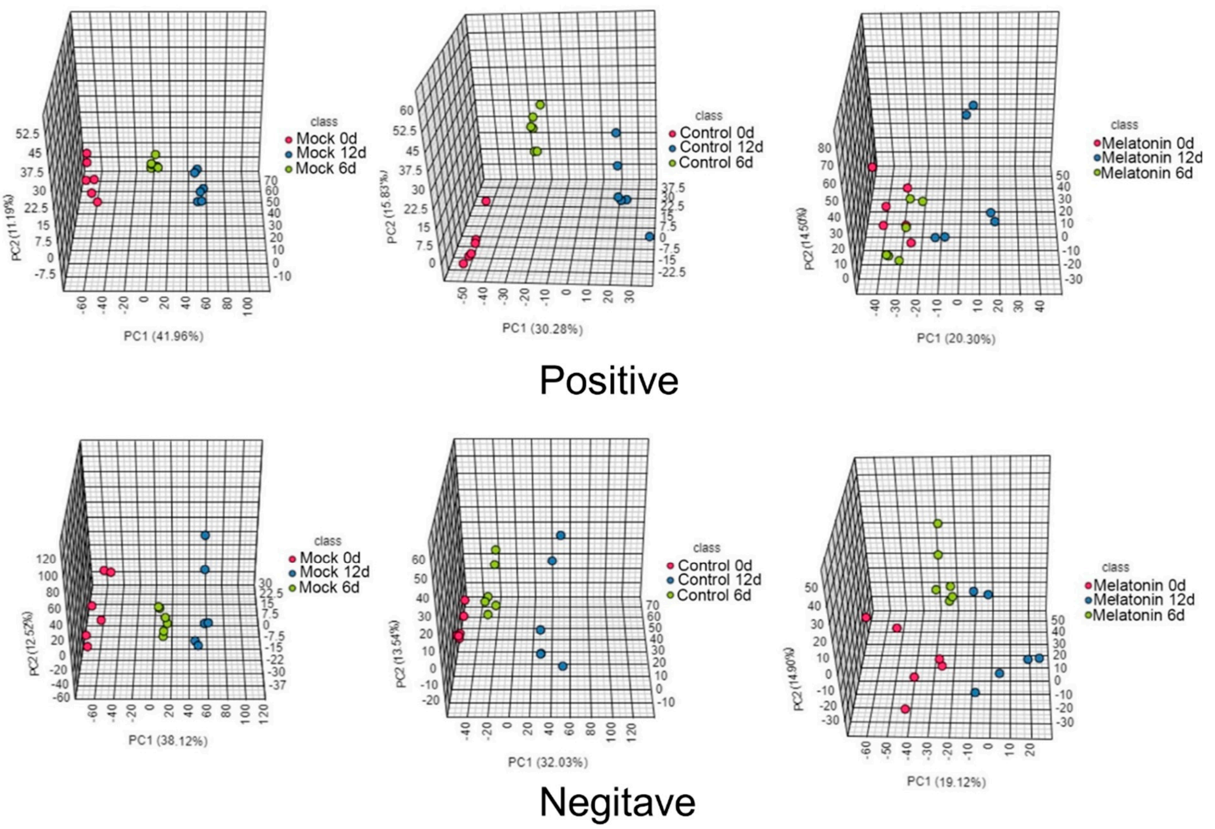

Figure S4. PLS-DA analysis of metabolomics changes of the fresh-cut lotus roots treated by exogenous melatonin.

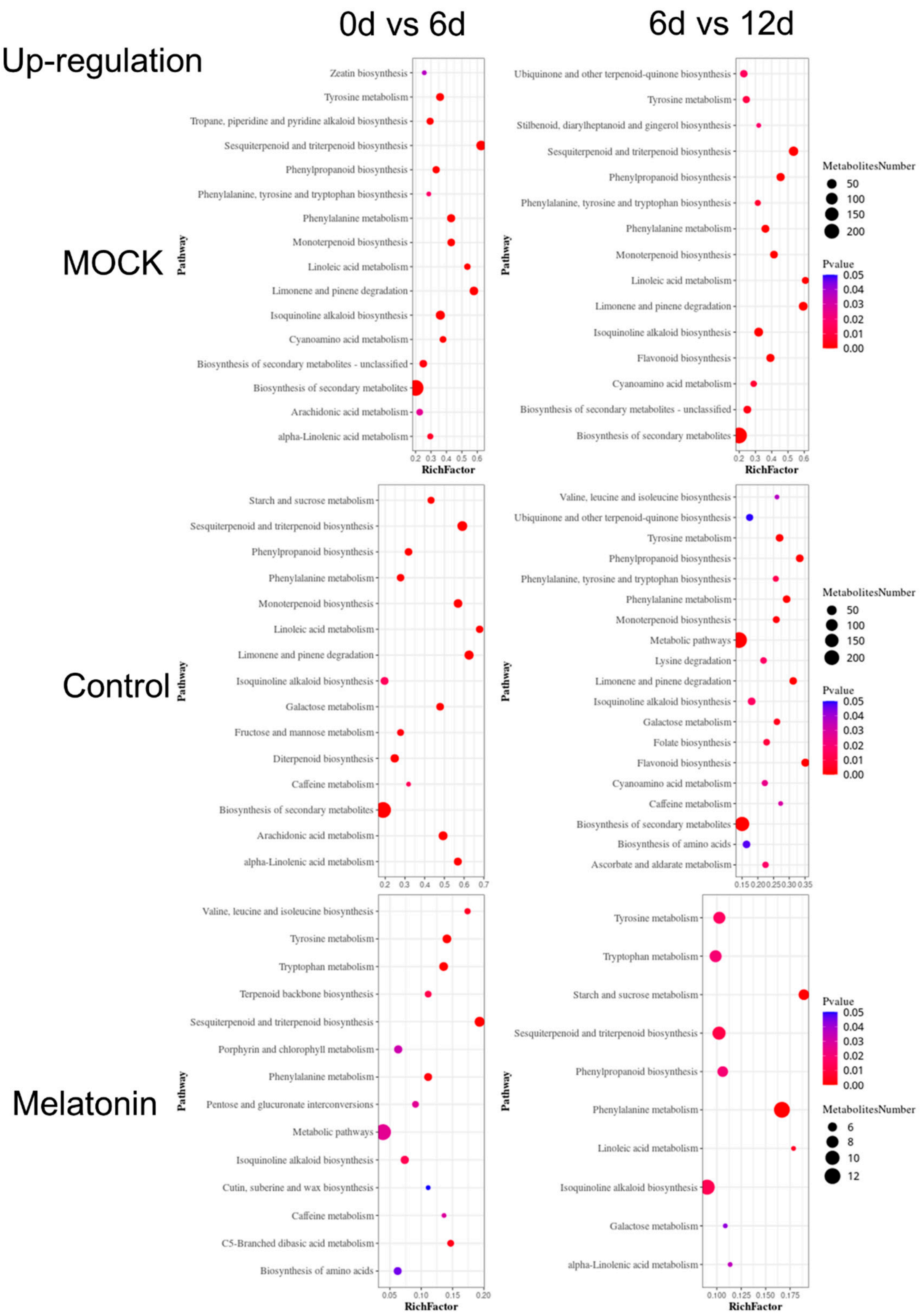

Figure S5. Differential metabolite pathway analysis of up-induced compounds in different treatment.

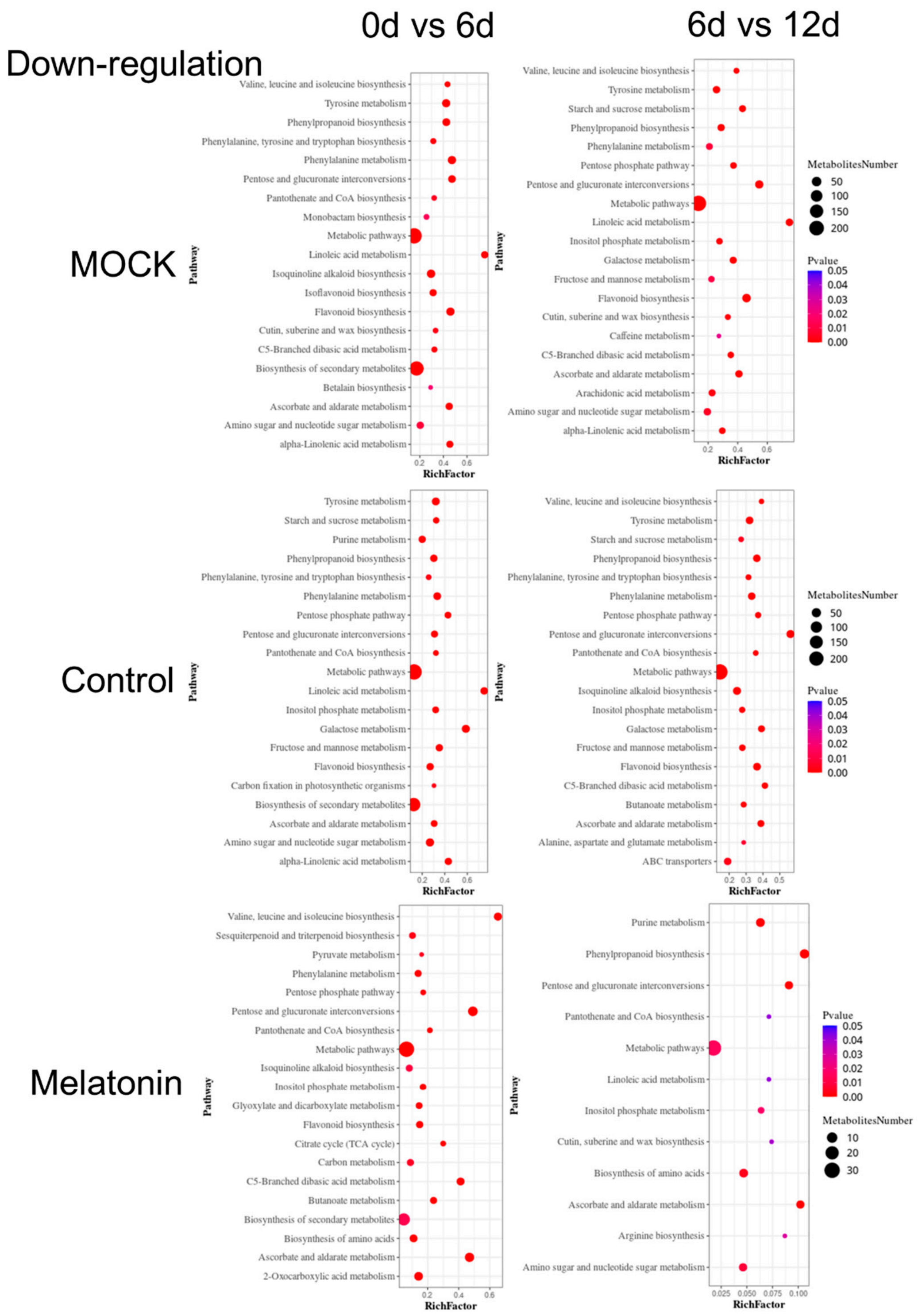

Figure S6. Differential metabolite pathway analysis of down-induced compounds in different treatment.

Table S1. Real-time PCR primers of genes.

Gene ID	Primers	Sequence (5'-3')
104611642	Forward:	CCCCAACGTCGAAACCAT
	Reverse:	CACAGCCCTGAACAAAGCA
104603071	Forward:	GGGAGTTGCTTTGGCTGTG
	Reverse:	TCCTATGCCGAATGTTGTGA
104599952	Forward:	CCATCTGTTCATCCTCTTGCTT
	Reverse:	TCGCTTCTGATAATGCTTTCC
104594740	Forward:	TATCGCCGTTAGGCTCTTGG
	Reverse:	GGTGGTGGCTCAGGTTTGTC
104608049	Forward:	ATGCCCTCCTCAAAATCAACT
	Reverse:	CCTGCCATCGTCCACTCA
104590242	Forward:	CTTGGGAAGGGAGGACATG
	Reverse:	ACAGCAGTAAGAGGTGGCAGA
104600124	Forward:	GCAGAGGCAGGTGCTTGA
	Reverse:	GCTCGTAGGGATTGGTGTCG
104592473	Forward:	CCGACCAAGAGCGACCAT
	Reverse:	AAATCCACGCCTGAACCTAA
104591893	Forward:	GTCTGTAGAGGAAGGGAGGGC
	Reverse:	AGCAGGAACAGCGGGGAG
104602250	Forward:	GGAGCAACAGTGGAAGAAGG
	Reverse:	TCAAAGTAGTCGGGGAAGGTC
β-Actin	Forward:	ACCACTGCTGAACGGGAAAT
	Reverse:	GGATGGCTGGAATAGAACCTCA
